# Cashew *(Anacardium occidentale* L.) Nuts Modulate the Nrf2 and NLRP3 Pathways in Pancreas and Lung after Induction of Acute Pancreatitis by Cerulein

**DOI:** 10.3390/antiox9100992

**Published:** 2020-10-14

**Authors:** Marika Cordaro, Roberta Fusco, Ramona D’Amico, Rosalba Siracusa, Alessio Filippo Peritore, Enrico Gugliandolo, Tiziana Genovese, Rosalia Crupi, Giuseppina Mandalari, Salvatore Cuzzocrea, Rosanna Di Paola, Daniela Impellizzeri

**Affiliations:** 1 Department of Biomedical, Dental and Morphological and Functional Imaging University of Messina, 98125 Messina, Italy; cordarom@unime.it; 2 Department of Chemical, Biological, Pharmaceutical and Environmental Sciences, University of Messina, 98166 Messina, Italy; rfusco@unime.it (R.F.); rdamico@unime.it (R.D.); rsiracusa@unime.it (R.S.); aperitore@unime.it (A.F.P.); egugliandolo@unime.it (E.G.); tgenovese@unime.it (T.G.); gmandalari@unime.it (G.M.); dimpellizzeri@unime.it (D.I.); 3 Department of Veterinary Sciences, University of Messina, 98168 Messina, Italy; rcrupi@unime.it; 4 Department of Pharmacological and Physiological Science, Saint Louis University School of Medicine, Saint Louis, MO 63104, USA

**Keywords:** cerulein-induced acute pancreatitis, cashew nuts, antioxidant, inflammation, polyphenols

## Abstract

Background: One of the most common co-morbidities, that often leads to death, associated with acute pancreatitis (AP) is represented by acute lung injury (ALI). While many aspects of AP-induced lung inflammation have been investigated, the involvement of specific pathways, such as those centered on nuclear factor E2-related factor 2 (Nrf2) and nucleotide-binding domain leucine-rich repeat (NLR) and pyrin domain containing receptor 3 (NLRP3), has not been fully elucidated. Methods: To investigate the effect of cashew (*Anacardium occidentale* L.) nuts on pancreatic and lung injury induced by cerulein injection, cerulein (50 μg/kg) was administered to CD1 mice for 10 h. Oral treatment with cashew nuts at a dose of 100 mg/kg was given 30 min and 2 h after the first cerulein injection. One hour after the final cerulein injection, mice were euthanized and blood, lung and pancreatic tissue samples were collected. Results: Cashew nuts were able to (1) reduce histological damage; (2) mitigate the induction of mast cell degranulation as well as the activity of myeloperoxidase and malondialdehyde; (3) decrease the activity levels of amylase and lipase as well as the levels of pro-inflammatory cytokines; and (4) enhance the activation of the Nrf2 pathway and suppress the activation of the NLRP3 pathway in response to cerulein in both pancreas and lung. Conclusions: Cashew nuts could have a beneficial effect not only on pancreatitis but also on lung injury induced by cerulein.

## 1. Introduction

Acute pancreatitis (AP) is a multifactorial disease, with a mortality rate that can be as high as 15–20%; it initiates in the pancreas in response to an inflammatory event and leads to deleterious local and systemic effects [1,2].

In particular, among the various organs that can be involved in the systemic inflammatory processes during pancreatitis, acute lung injury (ALI) is the most recurring severe complication and is actually the major cause of death in patients with AP [3,4]. While the detailed mechanisms of AP are still unknown, its pathogenesis is commonly attributed to the release of proteolytic enzymes, such as amylase and lipase, inflammatory elements, reactive oxygen species (ROS), peroxidation of lipid membranes, and release of other mediators into the blood, collectively leading to activation of the systemic inflammatory response [5,6,7,8].

Considering that the clinical course is extremely variable, research to discover new therapeutic approaches is extremely important [9]. The current treatment for pancreatitis is only supportive therapy, comprising primarily pain management, hydration and nutritional support. Given the multiple pathogenic mechanisms involved in AP and the associated systemic inflammatory reaction, one potentially useful approach is the identification of natural products with multiple modes of action, such as modulation of enzyme activities, suppression of inflammatory pathways, scavenging of free radicals, etc. [10]. Recent findings indicate that the nuclear factor E2-related factor 2 (Nrf2) and nucleotide-binding domain leucine-rich repeat containing family, pyrin domain-containing 3 (NLRP3) inflammasome pathways are strongly activated during the development of AP [10,11,12,13,14].

Nrf2 is a master regulator of protective antioxidant and anti-inflammatory responses. It coordinates the expression of several genes, including not only genes encoding antioxidant enzymes but also a series of genes involved in various processes including respiratory, cardiovascular, cerebrovascular, neurodegenerative and ocular diseases, as well as in tumorigenesis [15,16,17]. Under physiological conditions, Nrf2 is sequestered in the cytoplasm by its inhibitor Kelch-like ECH-associated protein 1 (Keap1), which mediates the proteasomal degradation of Nrf2 [18]. Once cellular oxidative stress occurs, Keap1 undergoes conformational modifications that prevent the degradation of Nrf2, allowing it to accumulate in the nucleus, where it activates the transcription of its target genes by binding to regulatory sequences called antioxidant response elements (ARE) [19]. Examples of antioxidant detoxification enzymes induced by Nrf2 include heme oxygenase 1 (HO-1) and manganese-dependent superoxide dismutase (Mn-SOD) [18].

Another fundamental pathway activated by the production of ROS is NLRP3 [20]. NLRP3 is part of the inflammasome, a multimeric protein complex comprising a sensor (NLRP3), an adaptor (apoptosis-associated speck like protein containing a caspase recruitment domain, ASC) and an effector (caspase 1), that initiates an inflammatory mode of cell death and triggers the release of pro-inflammatory cytokines [21]. The NLRP3 inflammasome has been implicated in a wide range of diseases, including AP, diabetes and prion and neurodegenerative diseases [22]. Studies have demonstrated that various natural compounds can ameliorate inflammation by inhibiting the NLRP3 pathway [23]. Natural compounds with the capacity to modulate the activation of NLRP3 may thus be considered as complementary treatments in acute and chronic inflammatory disorders. Such compounds include dietary antioxidants, such as curcumin, epigallocatechin-3-gallate (EGCG), mangiferin, and resveratrol [24]. To date, the use of cashew nuts (*Anacardium occidentale* L.), which are rich in antioxidants, has not been investigated as a possible strategy to counteract the development of inflammation in AP.

Cashew nuts represent a well-known medicinal plant with a powerful antioxidant and anti-inflammatory activity. They are rich in unsaturated fatty acids (UFAs), flavonoids, anthocyanins and tannins, fiber, folate, and tocopherols [25,26,27,28,29]. Different studies have consistently shown that adding nuts to a balanced diet helps to lose weight, lower cholesterol, control blood sugar, and protect the eyes, heart and skin. Cashew nuts have been recently used as treatment for several different diseases, both acute and chronic, such as colitis, joint degeneration, and dyslipidemia [30,31,32,33,34,35,36]. In the present study we evaluated the impact of oral treatment with cashew nuts on pancreas and lung during cerulein-induced AP in mice [37,38].

## 2. Materials and Methods

### 2.1. Animals

CD1 mice (25–30 g, Envigo, Milan, Italy) were employed. The University of Messina Review Board for animal care (OPBA) approved the study (protocol number 650/2017-PR dated 8/21/2017). All animal experiments were in compliance with the new Italian regulations (D.Lgs 2014/26), the EU regulations (EU Directive 2010/63) and the ARRIVE guidelines.

### 2.2. Experimental Protocol

AP was induced by cerulein hyperstimulation through 10 intraperitoneal (i.p.) injections (one injection every hour for 10 h at a dose of 50 μg/kg). Animals were euthanized one hour after the last injection, and samples of blood, lung, and pancreatic tissue were collected for further study [39].

### 2.3. Experimental Groups

Mice were randomly distributed into the following groups:

(1) Sham: Animals were subjected to injections of saline and were treated by oral gavage with saline.
(2) Sham + cashew nuts (100 mg/kg): Animals were subjected to injections of saline and were treated by oral gavage with cashew nuts at the dose of 100 mg/kg (data not shown because there were no differences between the sham+saline and sham+cashew nuts groups.).
(3) Cerulein: Mice were subjected to cerulein injections as described above and treated by oral gavage with saline.
(4) Cashew nuts (100 mg/kg): Mice were subjected to cerulein injections a described above and treated by oral gavage with cashew nuts (100 mg/kg).

The cashew nuts were given 30 min and 2 h after the first cerulein injection [1] (experimental timeline in Supplementary Figure S1). The dose used was chosen based on previous studies [31,32,36].

### 2.4. Pancreatic and Lung Oedema

Pancreatic and lung oedema was quantified as previously described by calculating the ratio between the water content of the tissue and its dry weight [40,41].

### 2.5. Histological Evaluation and Detection of Mast Cells

At the end of experiments, pancreas and lung tissues were fixed in 10% (*w/v*) PBS-buffered formaldehyde at room temperature. Seven micrometer sections were prepared from paraffin embedded tissues and stained with hematoxylin and eosin (H&E) for histological evaluation and with toluidine blue for detection of mast cells. After staining, they were evaluated using a Leica DM6 microscope (Leica Microsystems SpA, Milan, Italy) with Leica LAS X Navigator software (Leica Microsystems SpA). The injury score for both pancreas and lung was calculated as previously described [40,42].

### 2.6. Measurement of Lipase, Amylase and Pro-Inflammatory Citokynes

Blood was collected and centrifuged, and the supernatant was used for measurement of serum amylase and lipase activities using respective commercial kits (Cusabio, Houston, TX, USA and Abcam, Cambrige, UK) (Cat.# CSB-EL001689MO and CSB-E16930m, respectively) [39]. Additionally, the plasma levels of interleukin 1 beta (IL-1β), IL-6 and TNF-α were determined by enzyme-linked immunosorbent assay (ELISA) kits (eBioscience, San Diego, CA, USA) (Cat.# BMS6002, BMS603-2 and BMS607-3, respectively) as previously described [43].

### 2.7. Evaluation of Myeloperoxidas and Malonaldehyde

Myeloperoxidase (MPO) and malonaldehyde (MDA) levels were assessed as previously described in both pancreas and lung tissue. Briefly, after homogenization in respective specific buffers, absorbance was measured at 650 nm, using a spectrophotometer. Levels were expressed in milli-units per 100 milligram (mU/100 mg) of tissue [44,45,46].

### 2.8. Western Blot Analysis

Western blots were executed previously described [47]. The following specific primary antibodies were used: Anti-NRF-2 (sc-365949, 1:1000, Santa Cruz Biotechnology, Santa Cruz, CA, USA); anti-HO-1 (sc-136960, 1:1000; Santa Cruz Biotechnology, CA, USA); anti-Mn-SOD (sc-137254, 1:1000, Santa Cruz Biotechnology, CA, USA); anti-NLRP3 (sc-134306, 1:1000, Santa Cruz Biotechnology, CA, USA); anti-Caspase-1 (sc-56036, 1:1000, Santa Cruz Biotechnology, CA, USA); and anti-ASC (sc-514414, 1:1000, Santa Cruz Biotechnology, CA, USA). Primary antibodies were mixed in 1× PBS, 5% *w/v* non-fat dried milk, 0.1% Tween-20, and incubated at 4 °C, overnight. Afterwards, blots were incubated with peroxidase-conjugated bovine anti-mouse IgG secondary antibody or peroxidase-conjugated goat anti-rabbit IgG (1:2000, Jackson Immuno Research) for 1 h at room temperature. As loading controls, membranes were also incubated with antibodies against laminin (sc-376248, 1:1000; Santa Cruz Biotechnology, CA, USA) or GADPH (sc-47724, 1:1000; Santa Cruz Biotechnology, CA, USA). Signals were detected with enhanced chemiluminescence detection system reagent according to manufacturer’s instructions (Super-Signal West Pico Chemiluminescent Substrate, Pierce). The relative expression of the protein bands was quantified by densitometry with Bio-Rad ChemiDoc XRS software (ImageLab, v6.0.1) and standardized to β-actin levels. Images of blot signals were imported to analysis software (Image Quant TL, v2003).

### 2.9. Immunohistochemical Localization of NRF2, HO-1, Mn-SOD, Caspase-1, and ASC

Pancreas and lung sections were incubated with the following primary antibodies: Anti-NRF2 (sc-365949, 1:200, Santa Cruz Biotechnology, CA, USA); anti-HO-1 (sc-136960, 1:200, Santa Cruz Biotechnology, CA, USA); anti-Mn-SOD (sc-137254, 1:200, Santa Cruz Biotechnology, CA, USA); anti-NLRP3 (sc-134306, 1:200, Santa Cruz Biotechnology, CA, USA); anti-Caspase-1 (sc-56036, 1:200, Santa Cruz Biotechnology, CA, USA); and anti-ASC (sc-514414, 1:200, Santa Cruz Biotechnology, CA, USA), as previously described [48]. Sections were then incubated with the following secondary antibodies: Peroxidase-conjugated bovine anti-mouse immunoglobulin G (IgG) or peroxidase-conjugated goat anti-rabbit IgG (1:2000, Jackson Immuno Research, West Grove, PA, USA). Specific marking was revealed with a biotin-conjugated goat anti-rabbit IgG or biotin-conjugated goat anti-mouse IgG and avidin-biotin peroxidase complex (Vector Laboratories, Burlingame, CA, USA). Graphic presentation of densitometric analyses was performed Image J software (v1.52a) as previously described [49]. All immunohistochemical analyses were conducted by an observer without knowledge of the treatments.

### 2.10. Cashew Nuts Nutritional Composition

The cashew kernel samples (*Anacardium occidentale* L.) used were obtained from Ivory Coast; per 100 g they contained 5.40 g moisture, 22.46 g protein, 44.19 g total lipids, 4.48 g total dietary fibre, 30.95 g total sugars, 2.68 g ash, and 80.01 mg total phenols. The nutritional composition was analyzed according to the Association of Official Analytical Chemists (AOAC) Official Method as previously reported [50,51,52,53].

### 2.11. Reagents

All other materials were purchased from Sigma-Aldrich Co. Stock solutions were prepared in nonpyrogenic saline (0.9% NaCl, Baxter Healthcare Ltd., Thetford, Norfolk, UK).

### 2.12. Data Analysis

All values are expressed as mean ± standard error of the mean (SEM). For in vivo experiments, each group comprised 6 animals. For experiments involving histology, images shown are representative at least 3 independent experiments on tissue sections collected from all animals in each group. The results were analyzed by one-way ANOVA followed by a Bonferroni post-hoc test for multiple comparisons. A *p* value < 0.05 was considered significant. ^#^ *p* < 0.05 vs. cerulein; ^##^ *p* < 0.01 vs. cerulein; ^###^ *p* < 0.001 vs. cerulein; * *p* < 0.05 vs. sham; ** *p* < 0.01 vs. sham; *** *p* < 0.001 vs. sham.

## 3. Results

### 3.1. Effect of Cashew Nuts on Cerulein-Induced Oedema and Tissue Damage

**Figure 1:**
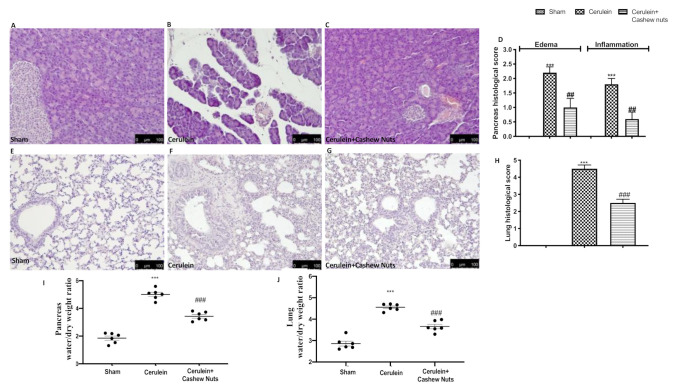
Evaluation of histological damage and oedema in the pancreas and lung of mice with cerulein-induced acute pancreatitis (AP). Pancreas: (**A**) sham, (**B**) cerulein, (**C**) cerulein+cashew nuts, (**D**) pancreas histological score, (**I**) pancreatic oedema. Lung: (**E**) sham, (**F**) cerulein, (**G**) cerulein + cashew nuts, (**H**) lung histological score, (**J**) lung oedema. Values shown are means ± SEM of 6 mice. *** *p* < 0.001 vs. sham; ^##^ *p* < 0.01 vs. cerulein; ^###^ *p* < 0.001 vs. cerulein.

Histological analysis of the pancreas of cerulein-treated mice showed tissue damage characterized by interstitial edema and inflammatory cell infiltrates (Figure 1B,D). These inflammatory signs were significantly reduced in the group of mice orally administered 100 mg/kg cashew nuts (Figure 1C,D). The histological analysis of the lung yielded similar findings. Lung injury during AP was characterized by alveolar thickening and abundance of inflammatory cell infiltrates (Figure 1F,H). Lung inflammation was significantly reduced by administration of cashew nuts (Figure 1G,H). Cerulein-induced AP is accompanied by tissue οedema in both the pancreas and the lung, which was quantified by determining the water content of the tissue. The oedema was significantly decreased after cashew nuts treatment in both pancreas (Figure 1I) and lung (Figure 1J).

### 3.2. Effects of Cashew Nuts on Cerulein-Induced Mast Cell Degranulation and on Myeloperoxidase and Malondialdehyde Activity

**Figure 2:**
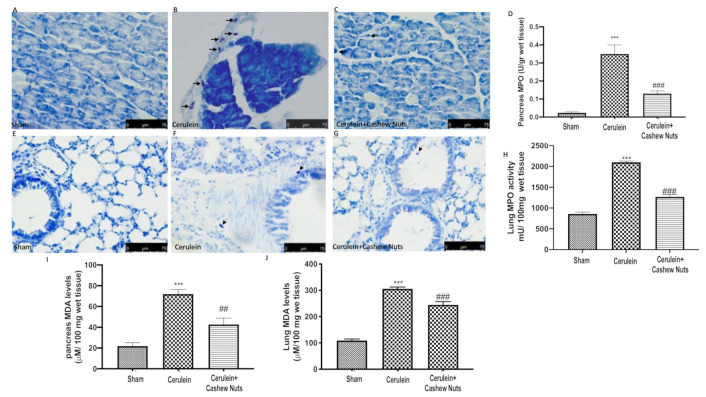
Effects of cashew nuts on cerulein-induced mast cell degranulation and MPO and MDA activity. Pancreas: (**A**) sham, (**B**) cerulein, (**C**) cerulein+cashew nuts, (**D**) MPO and (**I**) MDA. Lung: (**E**) sham, (**F**) Cerulein, (**G**) cerulein+cashew nuts, (**H**) MPO and (**J**) MDA. Arrows indicate mast cells. Values shown are means ± SEM of 6 mice. *** *p* < 0.001 vs. sham; ^##^ *p* < 0.01 vs. cerulein; ^###^ *p* < 0.001 vs. cerulein.

Mast cells are well known to play a significant role under inflammatory conditions, and there are remarkable overlaps between factors that cause mast cell degranulation and the progression of AP. We therefore evaluated whether administration of cashew nuts could have a beneficial effect on mast cell degranulation during cerulein-induced AP, as assessed by toluidine blue staining. In both pancreas (Figure 2B) and lung (Figure 2F), a significant increase in mast cell degranulation was observed after cerulein injection as compared to the sham group (Figure 2A,E). Treatment with cashew nuts significantly decreased the number of degranulated mast cells in pancreas (Figure 2C) and lung (Figure 2G). Moreover, oral treatment with cashew nuts significantly mitigated the cerulein-induced increase in the activity of malondialdehyde (MDA, a marker of lipid peroxidation) and myeloperoxidase (MPO, a marker of neutrophilic infiltration) in pancreas (Figure 2D,I) and lung tissue (Figure 2H,J).

### 3.3. Effects of Cashew Nuts on the Levels of Amylase, Lipase, and Pro-Inflammatory Cytokines

**Figure 3:**
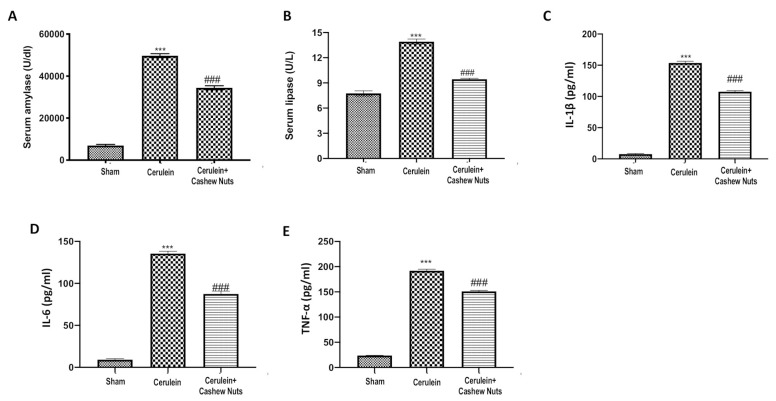
Effects of cashew nuts on the levels of amylase, lipase and cytokines in cerulein-induced AP. Amylase (**A**), lipase (**B**), IL-1β (**C**), IL-6 (**D**), and TNF-α (**E**). Values shown are means ± SEM of 6 mice. *** *p* < 0.001 vs. sham; ^###^ *p* < 0.001 vs. cerulein.

Administration of cerulein is well-known to cause an increase in the serum levels of amylase and lipase, as well as to promote the release of different pro-inflammatory cytokines into the blood. Indeed, in cerulein-induced AP, a significant increase in serum levels of amylase (Figure 3A), lipase (Figure 3B), IL-1β (Figure 3C), IL-6 (Figure 3D), and TNF-α (Figure 3E) was observed compared to the sham group; administration of cashew nuts significantly ameliorated all the above inflammatory markers.

### 3.4. Effects of Cashew Nuts on the Nrf2 Pathway in Cerulein-Induced AP

**Figure 4:**
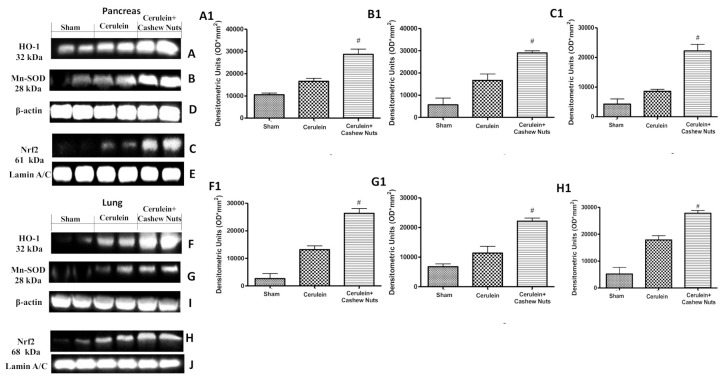
Effects of cashew nuts on the nuclear factor E2-related factor 2 (Nrf2) pathway in cerulein-induced AP as assessed by Western blotting. Pancreatic Western blots of HO-1 (**A**), manganese-dependent superoxide dismutase (Mn-SOD) (**B**), nuclear Nrf2 (**C**), β-actin (**D**) and Lamin A/C (**E**). Relative densitometric quantification of pancreatic HO-1 (A1), Mn-SOD (B1) and nuclear Nrf2 (C1). Lung Western blots of HO-1 (**F**), Mn-SOD (**G**), Nrf2 (**H**), β-actin (**I**) and Lamin A/C (**J**). Relative densitometric quantification of lung HO-1 (F1), Mn-SOD (G1 and nuclear Nrf2 (H1). Western blots shown are representative of at least 3 independent experiments. Values shown are means ± SEM of 6 mice. ^#^ *p* < 0.05 vs. cerulein.

Considering the key role of oxidative stress during AP, we investigated the effect of cashew nuts on the Nrf2 pathway in pancreas and lung by Western blotting. Administration of cashew nuts following cerulein injection significantly increased the nuclear protein abundance of Nrf2 in both pancreas (Figure 4C,C1) and lung (Figure 4H,H1). The gene encoding Mn-SOD is known to be upregulated by Nrf2, and cashew nuts significantly increased the protein abundance of Mn-SOD in both pancreas (Figure 4B,B1) and lung (Figure 4G,G1). The same pattern was observed for HO-1 in both pancreas (Figure 4A,A1) and lung (Figure 4F,F1).

**Figure 5:**
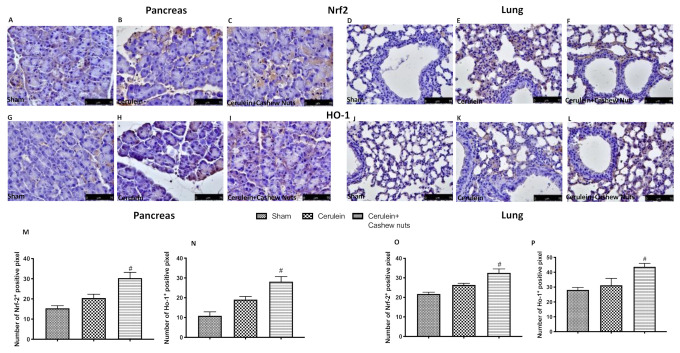
Effects of cashew nuts on the Nrf2 pathway in cerulein-induced AP as assessed by immunohistochemistry. Immunohistochemical staining for Nrf2 and HO-1 in pancreatic and lung tissue. Nrf2 immunohistochemical staining in pancreas ((**A**) sham, (**B**) cerulein, (**C**) cerulein+cashew nuts) and lung ((**D**) sham, (**E**) cerulein, (**F**) cerulein+cashew nuts), and relative densitometric quantification in pancreas (**M**) and lung (**O**). HO-1 immunohistochemical staining in pancreas ((**G**) sham, (**H**) cerulein, (**I**) cerulein+cashew nuts) and lung ((**J**) sham, (**K**) cerulein, (**L**) cerulein+cashew nuts) and relative densitometric quantification in pancreas (**N**) and lung (**P**). Values shown are means ± SEM of 6 mice. ^#^ *p* < 0.05 vs. cerulein.

These results were further confirmed by immunohistochemical staining for Nrf2 in pancreas (Figure 5A–C,M) and lung (Figure 5D–F,O), as well as for HO-1 in pancreas (Figure 5G–I,N) and lung (Figure 5J–L,P).

### 3.5. Effects of Cashew Nuts on the NLRP3 Pathway in Cerulein-Induced AP

**Figure 6:**
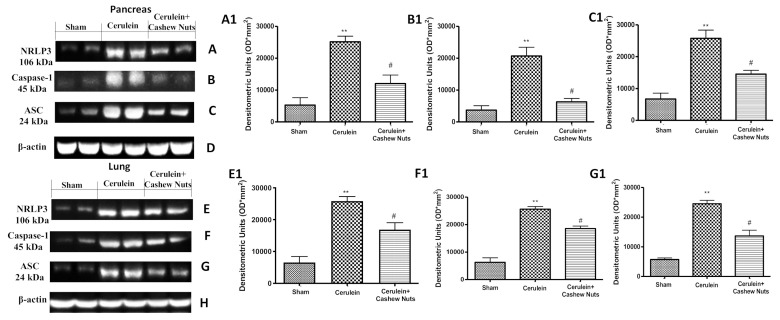
Effects of cashew nuts on the NLRP3 pathway in cerulein-induced AP as assessed by Western blotting. Pancreatic Western blots of NLRP3 (**A**), Caspase-1 (**B**), ASC (**C**) and β-actin (**D**). Relative densitometric quantification for pancreatic NLRP3 (A1), Caspase-1 (B1) and ASC (C1). Lung Western blots of NLRP3 (**E**), Caspase-1 (**F**), ASC (**G**) and β-actin (**H**). Relative densitometric analysis of lung NLRP3 (E1), Caspase-1 (F1) and ASC (G1). Values shown are means ± SEM of 6 mice. ^#^ *p* < 0.05 vs. cerulein; ** *p* < 0.01 vs. sham.

Finally, we evaluated whether cashew nuts could reduce inflammasome activation in cerulean-induced AP. Analysis of inflammasome components by Western blotting showed that the protein abundance of NLRP3 increased significantly in pancreas (Figure 6A,A1) and lung (Figure 6E,E1). The same was observed for the protein abundance of ASC in pancreas (Figure 6C,C1) and lung (Figure 6G,G1), as well as for the protein abundance of Caspase-1 in pancreas (Figure 6B,B1) and lung (Figure 6F,F1); administration of cashew nuts significantly ameliorated all of the above parameters.

**Figure 7:**
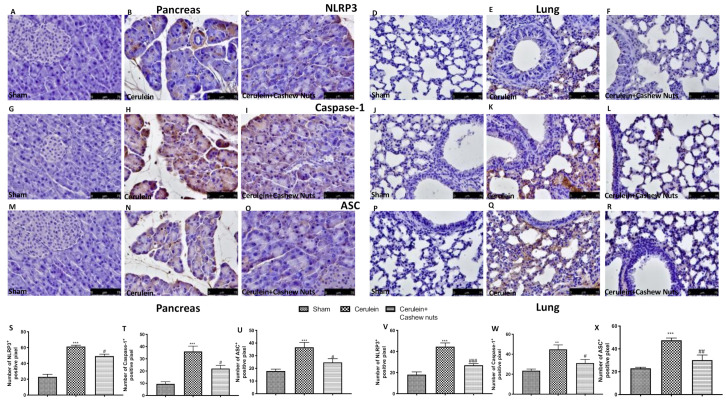
Effects of cashew nuts on the NLRP3 pathway in cerulein-induced AP as assessed by immunohistochemistry. Immunohistochemical staining for NLRP-3, Caspase-1, and ASC in pancreatic and lung tissue. NLRP3 immunohistochemical staining in pancreas ((**A**) sham, (**B**) cerulein, (**C**) cerulein+cashew nuts) and lung ((**D**) sham, (**E**) cerulein, (**F**) cerulein+cashew nuts), and relative densitometric quantification in pancreas (**S**) and lung (**V**). Caspase-1 immunohistochemical staining in pancreas ((**G**) sham, (**H**) cerulein, (**I**) cerulein+cashew nuts) and (lung (**J**) sham, (**K**) cerulein, (**L**) cerulein+cashew nuts), and relative densitometric quantification in pancreas (**T**) and lung (**W**). ASC immunohistochemical staining in pancreas ((**M**) sham, (**N**) cerulein, (**O**) cerulein+cashew nuts) and lung ((**P**) sham, (**Q**) cerulein, (**R**) cashew nuts), and relative densitometric quantification in pancreas (**U**) and lung (**X**). ^#^ *p* < 0.05 vs. cerulein; ^##^ *p* < 0.01 vs. cerulein; ^###^ *p* < 0.001 vs. cerulein; *** *p* < 0.001 vs. sham.

These results were further confirmed by immunohistochemical staining for NLRP3 in pancreas (Figure 7A–C,S) and lung (Figure 7D–F,V), as well as for Caspase-1 in pancreas (Figure 7G–I,T) and lung (Figure 7J–L,W), and for ASC in pancreas (Figure 7M–O,U) and lung (Figure 7P–R,X).

## 4. Discussion

AP is a common disease whose severity can vary mild disease to sepsis and multiple organ failure (MOF) [54]. Though AP can affect various distant organs, such as the colon, ALI is considered the most frequent possible complication of AP [41,55]. The relationship between AP and ALI is most probably due to an increase in the number of neutrophils in the lungs that lead to ROS generation with a consequent increase in the production of proinflammatory cytokines [56]. Human studies have indeed demonstrated very high concentrations of IL-1β, IL-6, TNF-α, neutrophil enzymes, and pancreatic enzymes including amylase and lipase in plasma, but the exact pathogenesis of AP-associated ALI remains unclear [56].

Even though there is still no specific drug therapy for AP, with treatment being generally supportive, it has been hypothesized that targeting inflammatory cascade molecules and oxidative stress could be a promising strategy to counteract the development of AP and ALI [57]. Some antioxidants, mainly naturally occurring ones, have been tested as potential beneficial agents in patients with AP. However, results to date have been inconsistent, and there are insufficient clinical data to support their routine use in humans. For example, resveratrol has been shown to be effective in the treatment of AP in rodent models, but clinical studies have not yet been conducted using this compound as an activator of Nrf2 [6]. In contrast, selenium, if given early, has been shown to reduce mortality, complications and need for surgery [58]. Intravenous administration of ascorbic acid (vitamin C) also significantly reduced markers of oxidative stress such as superoxide dismutase and catalase, and led to a faster normalization of the leukocyte count and of amylase levels, as well as to a significant reduction of TNF-α, IL-6, and IL-8 levels [59]. Melatonin has also been shown to be able to neutralize oxygen radicals, activate enzymes involved in the antioxidant response, and suppress the release of pro-inflammatory cytokines [60].

Despite such promising results, the data currently available are not sufficient to support the clinical use of antioxidants for AP. Therefore, further studies are needed to understand the precise mechanisms underlying this serious disease and to optimize its treatment by counteracting both the inflammatory and oxidative processes implicated in its pathogenesis. In this regard, several trials in recent years have focused on the use of nutritional support to traditional treatment [61].

Cashew nuts, fruits of *Anacardium occidentale* L., an original plant from Brazil, have shown a good capacity to counteract oxidative damage, primarily thanks to the abundance of secondary metabolites such as polyphenols, flavonoids and others [62,63,64,65,66]. The most plausible hypothesis is that polyphenolic components of dietary plants modulate the cellular redox state by boosting the endogenous antioxidant defense [66,67]. Recently, cashew nuts were used for their antioxidant, anti-genotoxic, anti-mutagenic, anti-inflammatory, and other protective properties [31,68,69,70,71,72,73,74,75]. In previous studies, we demonstrated that cashew nuts treatment, was able to alleviate oxidative stress and inflammation in different in vivo models, such as dinitrobenzene sulfonic acid (DNBS)-induced colitis, carrageenan-induced paw edema, and monosodium iodoacetate (MIA)-induced osteoarthritis. These effects are likely exerted through a reduction of various pro-inflammatory pathways and mediators, including MPO and MDA levels, mast cell degranulation and neutrophil infiltration, release of pro-inflammatory cytokines, modulation of NF-κB signaling, modulation of ROS production, etc. [31,32,36].

Among several different in vivo experimental models of AP that exhibit the same pathophysiological development of human pancreatitis, the use of cerulein, an analog of cholecystokinin (CCK), is one of most frequently used. We thus used this model to induce AP in mice and to investigate for the first time the effect of cashew nuts treatment on inflammation and oxidative stress in the pancreas and lung during AP.

Cashew nuts treatment had beneficial effects on cerulein-induced histological alterations in both pancreas and lung. Cerulein treatment led to severe alterations of tissue architecture with oedema formation and inflammatory cells infiltration. These modifications were significantly attenuated by oral treatment with cashew nuts at a dose of 100 mg/kg. Mast cells have been reported to play a pivotal role during pancreatitis-associated ALI [76]. Previous studies have shown that mast cells are usually located in the pancreatic interstitial and periacinar space as well as in the mesentery, but during AP they were highly correlated with neutrophil infiltration and oedema formation in both pancreas and lung [76,77]. Neutrophil infiltration is also important in acute pancreatitis. MPO activity is a useful indicator of neutrophil activation and inflammation, since the enzyme is stored in the neutrophils’ granules [78]. Additionally, because while one of the most dangerous consequences of oxidative stress is cellular injury triggered by ROS, it is informative to assess the levels of oxidation products as markers of oxidative stress [79,80]. Considering that lipid peroxides are extremely reactive compounds, they degrade rapidly into a range of metabolites. MDA is one of the best known secondary metabolites of lipid peroxidation, and it is used as a marker of cell membrane damage [80]. In the present study, oral treatment with cashew nuts was able to partially suppress mast cell degranulation, neutrophil infiltration, and lipid peroxidation.

Cerulein administration is well known to induce a dysregulation of the production and secretion of digestive enzymes, such as amylase and lipase, specifically inhibiting their secretion by the exocrine pancreas into the digestive tract, and leading to elevation in their respective levels in the blood circulation [81]. In parallel, pro-inflammatory cytokines play a fundamental role in the inflammatory response associated with AP. Different clinical studies have, in fact, documented a pro-inflammatory cytokine profile in the sera of patients with AP, including increased levels of IL-1β, IL-6 and TNF-α [82,83]. This profile was also observed in the present study. Importantly, cashew nuts were able to decrease the cerulein-induced levels of amylase and lipase as well as the levels of IL-1β, IL-6 and TNF-α.

AP being an oxidative stress condition, the Nrf2/Keap1 signaling pathway is activated in the pancreas, but this not sufficient to prevent the disease [12]. Several studies have shown that the hyper-stimulation of Nrf2 via plant-derived natural compounds such as visnagin or hydroxytyrosol could be a promising strategy against the excessive oxidative stress that characterizes AP [41,84,85]. In the present study, we found that cashew nuts treatment was able to promote Nrf2 nuclear translocation and to induce the expression of the Nrf2-regulated factors HO-1 and Mn-SOD in both pancreas and lung.

Another pathway that recently attracted attention is NRLP3, and it has been recently demonstrated that NLRP3 modulation may be a promising strategy to alleviate AP and ALI [86,87]. Inflammasome formation starts with the interaction of NRLP3 with ASC, which in turn recruits and activates procaspase-1 to active caspase-1, converting the cytokine precursors pro-IL-1β and pro-IL-18 into mature and active IL-1β and IL-18, respectively. The activation of these cytokines leads to a series of cellular responses that induce a very strong inflammatory response in the cell which can culminate in its death [88,89,90,91,92,93]. Researchers have focused their attention on the role of the inflammasome in the initiation or evolution of disorders with a high impact on public health, such as metabolic pathologies, cardiovascular diseases, inflammatory issues, and neurologic disorders [20]. It has been shown that the NLP3-induced caspase-1-mediated activation and secretion of IL-1β and IL-18 plays a key role during the development of AP [11]. In the present study, we found that the levels of NLRP3, ASC and caspase-1 were significantly increased after cerulein induction, and that cashew nuts considerably diminished this increase in both pancreas and lung.

## 5. Conclusions

Considering the key role played by inflammation and oxidative stress in several diseases, antioxidant and anti-inflammatory dietary compounds are a main research attention is focus. Antioxidant treatment is believed to have great prospects, since its therapeutic efficacy has already been demonstrated in several experimental settings of AP. Nuts are one of the main sources of polyphenols in the diet worldwide. The present work adds further support to the concept that natural-based compounds can be useful for the treatment not only of pancreatitis but also of the lung complications associated with it. Specifically, compounds present in cashew nuts could be a useful adjunct to mitigate the inflammation and oxidative stress that underlie these conditions.

## Supplementary Materials

The following are available online at https://www.mdpi.com/2076-3921/9/10/992/s1, Figure S1: Experimental protocol of cerulein-induced acute pancreatitis (AP). AP was induced by cerulein hyperstimulation through ten hourly intraperitoneal (i.p) injection at the dose of 50 μg/kg. Cashew nuts were given 30 min and 2 h after the first cerulein injection. Animals were euthanized 1 h after the last injection, and samples of blood, lung and pancreatic tissue were preserved for further study.
