# Supplementary material for: Cashew (Anacardium occidentale L.) Nuts Modulate the Nrf2 and NLRP3 Pathways in Pancreas and Lung after Induction of Acute Pancreatitis by Cerulein
Source: Antioxidants (Basel). 2020 Oct 14;9(10):992. doi: 10.3390/antiox9100992 (PMC7602264; doi:10.3390/antiox9100992)
Supplement: Supplementary file 1 [file antioxidants-09-00992-s001.pdf]

antioxidants

Supplementary materials

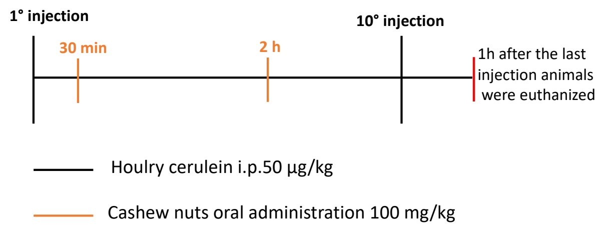

Figure 1. Experimental protocol of cerulein-induced acute pancreatitis (AP). AP was induced by cerulein hyperstimulation through ten hourly intraperitoneal (i.p) injection at the dose of 50 µg/kg. Cashew nuts were given 30 min and 2 h after the first cerulein injection. Animals were euthanized 1 h after the last injection, and samples of blood, lung and pancreatic tissue were preserved for further study.
